# Distribution of Coronavirus Receptors in the Swine Respiratory and Intestinal Tract

**DOI:** 10.3390/vetsci9090500

**Published:** 2022-09-13

**Authors:** Rahul Kumar Nelli, James Allen Roth, Luis Gabriel Gimenez-Lirola

**Affiliations:** College of Veterinary Medicine, Iowa State University, Ames, IA 50010, USA

**Keywords:** ACE2, DPP4, APN, CEACAM1, coronavirus receptors, intestines, trachea, lung, swine

## Abstract

**Simple Summary:**

Overall, viral receptors are cell surface molecules that allow for virus binding and cell entry leading to productive infection. The presence and distribution of these receptors across tissues can determine susceptibility and the outcome of the infection. This study demonstrates the presence of the main protein receptors used by different coronaviruses in the respiratory and intestinal tract of pigs. The present study has important implications for the development of research models and for the assessment of the potential risk and introduction of coronaviruses into the swine population.

**Abstract:**

Coronaviruses use a broad range of host receptors for binding and cell entry, essential steps in establishing viral infections. This pilot study evaluated the overall distribution of angiotensin-converting enzyme 2 (ACE2), aminopeptidase N (APN), carcinoembryonic antigen-related cell adhesion molecule 1 (CEACAM1), and dipeptidyl peptidase 4 (DPP4) receptors in the pig respiratory and intestinal tract. All the receptors evaluated in this study were expressed and differentially distributed through the respiratory and intestinal tract. The presence and expression levels of these receptors could determine susceptibility to coronavirus infections. This study may have important implications for the development of research models and the assessment of the potential risk and introduction of novel coronaviruses into the swine population.

## 1. Introduction

Coronaviruses (CoVs) are enveloped, single-stranded positive-sense RNA viruses that infect a wide variety of mammals and birds, causing predominantly respiratory and enteric diseases [1,2]. CoVs use a broad range of host receptors for binding (viral spike proteins) and cell entry, which are essential steps in establishing viral infections [3]. The distribution of these receptors among tissues and across animal species partially determines tissue/cell tropism and the host range of specific CoVs. The presence of CoV-specific receptors does not necessarily indicate susceptibility to infection. Knowledge of viral spike proteins and cell receptors is essential for the rational development of antiviral interventions (receptor-blocking drugs and vaccines), mainly for the recently re/emerged CoVs, e.g., porcine epidemic diarrhea virus (PEDV), porcine delta coronavirus (PDCoV), Middle East respiratory syndrome-related coronavirus (MERS-CoV), and severe acute respiratory syndrome-related coronavirus-2 (SARS-CoV-2) (Table 1). Pigs are susceptible to different CoVs belonging to all genera, with the exception of *Gammacoronaviruses* [4]. However, comprehensive studies evaluating the distribution of CoV receptors and their implications for the susceptibility of pigs to infection are scarce. These studies have important implications for the development of in vivo (“bioassay”) and ex vivo (“organoids”) research models and for the assessment of the potential risk of introduction of novel CoVs into the swine population.

## 2. Materials and Methods

### 2.1. The Study

This pilot study evaluated the distribution of the primary glycoprotein receptors for CoVs, i.e., angiotensin-converting enzyme 2 (ACE2) [5], aminopeptidase N (APN) [6], carcinoembryonic antigen-related cell adhesion molecule 1 (CEACAM1) [7], and dipeptidyl peptidase 4 (DPP4) [8] in tissue sections of the trachea, lung, small intestine (duodenum, jejunum, ileum), and large intestine (cecum, colon, and rectum) of pigs. Sequence analysis (BLAST) showed that the homology of these receptors in pigs ranged from 55.5% to 88.3%, identical to the human receptors at the amino acid level. These multi-functional molecules are expressed in endothelial cells, epithelial cells, and immune cells of different animal species, and the modulation of their expression is critical for several physiological and pathological processes [9,10]. All samples were collected following according to the Institutional Animal Care and Use Committee (IACUC; log number 12-17-8658-S; approval date, 3 January 2018) approval by the Iowa State University (ISU).

### 2.2. Sample Collection

Tissues were dissected aseptically from 7–10 days-old CD/CD pigs (Yorkshire × Large White crossbred) immediately after necropsy and were either snap-frozen in liquid nitrogen and stored at −80 °C or fixed in 10% buffered neutral formalin for gene or protein expression analysis, respectively. 

### 2.3. Quantitative PCR

The constitutive mRNA levels of *ACE2, CEACAM, DPP4,* and *APN* in selected tissues were determined by qPCR. Snap-frozen swine tissues were homogenized in TRIzol reagent (Thermo Fisher Scientific, Waltham, MA, USA) using 2.8 mm ceramic beads (Omni). Homogenates were subjected to chloroform phase separation, and total RNA was extracted from the clear aqueous phase following the manufacturer’s instructions (QIAGEN, Germantown, MD, USA). Total RNA concentration and purity were evaluated using NanoDrop one microvolume UV-Vis spectrophotometer (Thermo Fisher Scientific). Samples with A260/280 between 1.96 and 2.05 were used for reverse transcription (150 ng total RNA) using a qScript XLT cDNA SuperMix (Quantabio, Beverly, MA, USA). qPCR reactions were performed using 1.5 ng/μL of cDNA, 1x PowerUp SYBR Green Master Mix (Thermofisher Scientific), and 500 nM of swine-specific primers (Table 1) in an Applied Biosystems 7500 Fast real-time system under these conditions: 50 °C for 2 min and 95 °C for 2 min holding; 40 cycles, 95 °C for 15 s denaturation and 60 °C for 1 min amplification; final melting curve analysis was performed at 95 °C for 15 s, 60 °C for 1 min and 95 °C for 15 s. qPCR reactions were performed in duplicate, including no-template controls. Amplification efficiencies beyond the range (1.90–2.2) and a threshold cycle above 35, including samples with multiple melting peaks, were discarded. 

### 2.4. Immunohistochemistry

**Figure 1:**
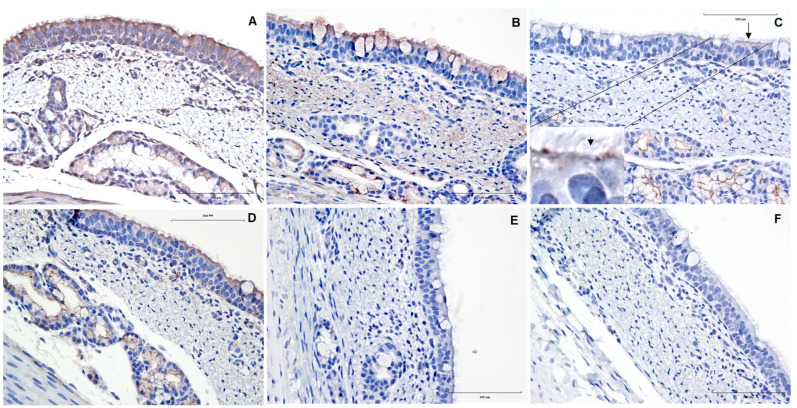
Immunohistochemically stained images of corona viral receptors in tracheal sections of pigs. Representative images of formalin-fixed paraffin-embedded cross-sections of pig trachea stained with ImmPRESS VR anti-mouse IgG horseradish peroxidase (HRP) polymer detection kit (MP-6402-15; Vector Laboratories) with mouse monoclonal antibodies (1:50) specific for (**A**) aminopeptidase N (APN/CD13; sc-166105, Santa Cruz); (**B**) dipeptidyl peptidase 4 (DPP4/CD26; CACT114A-BOV2078 (The Washington State University Monoclonal Antibody Center, Pullman, WA, USA); (**C**) angiotensin-converting enzyme 2 (ACE2; sc-390851, Santa Cruz Biotechnology, Dallas, TX, USA)—note the focal expression of ACE2 on epithelial cells (arrow); (**D**) carcinoembryonic antigen-related cell adhesion molecule 1 (CEACAM1/CD66a; sc-166453, Santa Cruz) receptors; (**E**) secondary antibody control and (**F**) blank. Brown represents a positive expression of the antibody, and the nucleus counterstained with hematoxylin is blue. Scale bar—100 μm.

Protein expression was assessed in paraffin-embedded tissue sections (5 μm) by IHC using primary antibodies against the different receptors (Figure 1). ImmPRESS VR anti-mouse IgG HRP-conjugated polymer detection kit (Vector Laboratories Inc., Burlingame, CA, USA) was used for secondary antibody staining following the manufacturer’s instructions. Deparaffinized sections were heat retrieved (96 °C/30 min) using citrate buffer (Millipore-Sigma, Burlington, MA, USA) and washed in tris-buffered saline containing 0.1% Tween 20 (TBST) (Millipore-Sigma, Burlington, MA, USA). After blocking with animal-free buffer (Vector Laboratories) for 30 min, sections were incubated overnight with the corresponding primary antibody at 4 °C. The sections were then treated with 0.3% hydrogen peroxide for 30 min, followed by incubation with the secondary antibody for 60 min. Chromogenic detection of sections was performed using ImmPACT DAB EqV peroxidase substrate solution (Vector Laboratories) and hematoxylin, followed by mounting in Tissue-Tek Glas mounting medium (Sakura Finetek Inc, Torrance, CA, USA).

## 3. Results and Discussion

A summary of the gene (RT-PCR) and protein (IHC) expression patterns of different coronavirus protein receptors in the pig respiratory and intestinal tract are presented in Table 1. All receptors were detected at some level in tissues of at least one piglet. *APN* gene and protein expression were detected in all tissues and pigs evaluated, particularly in the epithelium and sub-epithelial regions of the trachea (Figure 1A), bronchiole, and lung. Previous studies reported APN expression in the small intestine of the swine fetus along the brush border and apical cytoplasm [11]. However, in the present study, APN expression was more uniform from the crypt region to the apical tip of the intestinal villi (image not shown).

Likewise, DPP4 was widely distributed across the respiratory and small intestinal tract; however, an irregular distribution was found in the large intestinal tract. Along with its expression in both the pseudostratified and subepithelial regions of the trachea, DPP4 was also observed inside the mucus-secreting goblet cells (Figure 1B). These findings are similar to previous studies in pigs, where DPP4 was expressed not only in the epithelial lining of the trachea but also in neutrophils and macrophage-like cells in the subepithelial region [12]. Studies in mice demonstrated DPP4 activity along the intestinal tract, but it was higher in the small intestine than in the large intestine [13]. However, no differences were noticed in DPP4 expression on porcine intestines.

**Figure 2:**
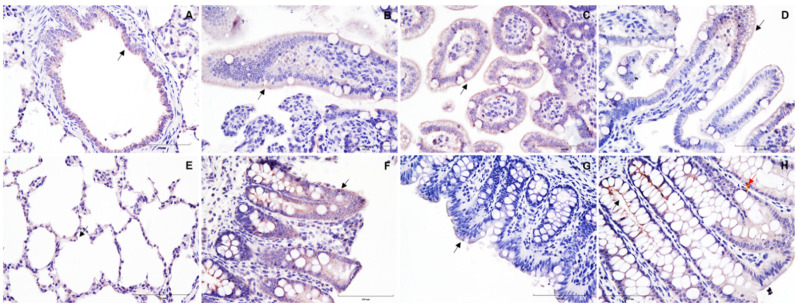
Immunohistochemically stained images of ACE2 receptors in the respiratory and intestinal tract of pigs. Representative images of formalin-fixed paraffin-embedded cross-sections of pig bronchiole (**A**); duodenum (**B**); jejunum (**C**); ileum (**D**); lung (**E**); cecum (**F**); colon (**G**); and rectum (**H**). Note the tissue marking dye artifact (red arrow). Brown represents a positive expression of the antibody (indicated by black arrows), and the nucleus counterstained with hematoxylin is blue. Scale bar—100 μm.

This is the first study reporting the section-specific expression patterns of ACE2 and CEACAM1 across the swine respiratory and intestinal tracts. ACE2 expression in the trachea was predominantly observed on the epithelial lining of submucosal glands, with an intermittent focal expression on the epithelial cells (Figure 1C). However, the expression of ACE2 became more distinct on the bronchiolar epithelial surface and the alveolar pneumocytes of the lung (Figure 2A,E). Similarly, other studies [14] found limited detection of ACE2 mRNA in pig lungs. It is important to recognize that there are dissenting studies on the presence and distribution of ACE2 receptors in the porcine respiratory tract. In 2021, a lack of ACE2 protein expression was reported by Lean and others [15] in the pig respiratory tract, while Di Teodoro and others [16] reported this finding in an ex vivo porcine respiratory culture system. It is noteworthy that in both these studies, a rabbit anti-ACE2 polyclonal antibody (Abcam) was used for IHC. However, a monoclonal ACE2 antibody (Santa Cruz) was used in the present study, yet different staining protocols were also used. All these factors may contribute to discrepant results between different studies.

Regarding the intestinal tract, *ACE2* gene and protein expression was broadly detected in different sections of the porcine small intestine. In this line, studies have shown that the expression of ACE2 protein was ubiquitous in the small intestine of different domestic, livestock and wild animal species, including pigs, via immunolabelling, particularly on the brush border of the epithelial cells and the tip of the small intestinal villi [15]. However, they did not perform section-specific analysis during this study. Interestingly, *ACE2* gene expression was not detected in the cecum and rectum regions of the large intestine, while only one pig (1/3) showed *ACE2* expression in the colon (Table 1). On the contrary, IHC analysis showed expression of ACE2 protein on the villi of both the cecum and rectum but not in the colon (Figure 2F–H). Its absence in the swine colon contrasts with previous studies reporting ACE2 expression in the human colon [17], suggesting differences in the distribution in closely related mammalian species (i.e., human versus pig) [15,16].

CEACAM1 was uniformly distributed across the pseudostratified epithelia and the submucosal glands of the trachea (Figure 1D), as well as the bronchiolar epithelium and alveolar pneumocytes (image not shown). In the intestine, gene expression patterns and distributions for *CEACAM1* were variable among the intestinal regions (e.g., no gene expression in the jejunum). However, IHC analysis revealed that CEACAM1 protein expression was primarily confined to the epithelial brush border (Table 1). A similar distribution pattern was also reported in the cancerous tissue of the human gastric mucosa [18].

It is important to highlight that in the absence of commercially available porcine antibodies, human monoclonal antibodies were used for ACE2, APN, and CEACAM1, while a bovine-specific monoclonal antibody was used for DPP4. However, with the exception of CEACAM1, which showed the lowest homology in the amino acid sequence (55.5% identity of swine (XP_020950079.1) with human (NP_001703.2)), the remaining receptors had an amino acid homology of ~80% or higher; for APN (78.8% between swine (NP_999442.1) and human (XP_011519775.1)); ACE2 (81.3% between swine (NP_001116542.1) and human (NP_001358344.1)); and DPP4 (88.3% between swine (NP_999422.1) and human (NP_001926.2), and 90% swine versus bovine (NP_776464.1)). Nevertheless, it is important to remember that antibody-based cross-reactivity strongly depends on a highly conserved conformational structure rather than the percentage of amino acid sequence.

This was a pilot study involving a relatively low number of animals, which could indeed be seen as a limitation. It is generally assumed that gene (mRNA) expression should result in the expression of its protein product. Certainly, the expression of a gene and the corresponding receptor protein provides robust evidence of its presence within a specific tissue. This is what we observed with all protein receptors in the respiratory tract and APN receptors in different respiratory and intestinal tissues. However, it is known that many genes and proteins may not be constitutively expressed across tissues but are subject to differential expression in response to a varied stimulus (i.e., biological meaning) [19]. Differentially expressed genes should correlate better with their product (protein) under specific circumstances. Moreover, receptor binding or mRNA expression can vary depending on the type and status of the sample, age, and gender of animals from which the samples are collected. Therefore, it would be interesting to assess the differential expression of these receptors in response to exposure or infection with different coronavirus [11,12,13].

## 4. Conclusions

Viral receptors are major drivers in defining the host range and specific tissue or cell type tropism of viruses. All CoV receptors evaluated in this study were expressed and differentially distributed through the respiratory and intestinal epithelia of swine. The presence and expression levels of specific receptors could determine the outcome of CoV infections. However, although mammalian CoV receptors are significantly conserved, only host-specific receptors can serve as efficient virus receptors [20]. Further research is needed to determine if the various coronaviruses bind to the swine receptors. 

## Figures and Tables

**Table 1:** Expression pattern of coronavirus receptors in the respiratory and intestinal tracts of 7- to 10-day-old pigs.

	Trachea	Bronchiole	Lung	Duodenum	Jejunum	Ileum	Cecum	Colon	Rectum	
**Immunohistochemistry**										**Viruses Showing Affinity to These Receptors**
APN/CD13	+ (2/2)	+ (2/2)	+ (2/2)	+ (3/3)	+ (3/3)	+ (3/3)	+ (3/3)	+ (3/3)	+ (3/3)	FCoV, CCoV, TGEV, PRCV, PEDV, PDCoV
DPP4/CD26	+ (2/2)	+ (2/2)	+ (2/2)	+ (3/3)	+ (3/3)	+ (3/3)	+ (2/3)	+ (2/3)	+ (2/3)	MERS-CoV
ACE2	+ ** (2/2)	+ (2/2)	+ (2/2)	+ * (3/3)	+ * (3/3)	+ * (3/3)	+ * (2/3)	− (3/3)	+ (2/3)	HCoV-NL63, SARS-CoV, SARS-CoV2
CEACAM1/CD66a	+ (2/2)	+ (2/2)	+ (2/2)	+ ^#^ (3/3)	+ ^#^ (3/3)	+ ^#^ (3/3)	+ ^#^ (2/3)	+ ^#^ (2/3)	+ ^#^ (2/3)	MHV
***Gene expression*** *(NCBI reference)*										**Primer sequences**
***APN/CD13*** (NM_214277.1)	+ (2/2)	Not performed	+ (2/2)	+ (3/3)	+ (3/3)	+ (3/3)	+ (3/3)	+ (3/3)	+ (3/3)	For Primer 5′-CACGACACAGATGCAGTCTACAGA-3′ Rev Primer 5′-TGTTGAACGTGGCCTTCATG-3′
***DPP4/CD26*** (NM_214257.1)	+ (2/2)	Not performed	+ (2/2)	+ (3/3)	+ (3/3)	+ (3/3)	+ (1/3)	+ (2/3)	+ (2/3)	For Primer 5′-ACCAGGACTCTCAGCCCAAA-3′ Rev Primer 5′-ACAAGTAGTGATCCCCTATTAACACAGA-3′
***ACE2*** (NM_001123070.1)	+ (2/2)	Not performed	+ (2/2)	+ (2/3)	+ (2/3)	+ (3/3)	− (3/3)	+ (1/3)	− (3/3)	For Primer 5′-GGGTGGTGATGGGATTGGTA-3′ Rev Primer 5′-TTGCTTTTTCTTCCTTCGATCTCT-3′
***CEACAM1/CD66a*** (XM_021094420.1)	+ (2/2)	Not performed	+ (2/2)	+ (2/3)	− (3/3)	+ (2/3)	+ (1/3)	+ (3/3)	+ (3/3)	For Primer 5′-TGCTCGCAGAGAGGATAAAACTG-3′ Rev Primer 5′-GGCCTCGCACTGATAATTCC-3′

The presence and absence of the receptors are represented by + and −, respectively. Values in parentheses were the number of pig tissues tested positive per total pig tissues tested. Aminopeptidase N (APN); angiotensin-converting enzyme 2 (ACE2); carcinoembryonic antigen-related cell adhesion molecule 1 (CEACAM1); dipeptidyl-peptidase 4 (DPP4); cluster of differentiation (CD); feline coronavirus (FCoV); canine coronavirus (CCoV) serotype 2; transmissible gastroenteritis virus (TGEV); porcine epidemic diarrhea virus (PEDV); severe acute respiratory syndrome-related coronavirus (SARS-CoV); mouse hepatitis virus (MHV); Middle East respiratory syndrome-related coronavirus (MERS-CoV); forward (For); reverse (Rev). ** Expression on the epithelial lining of submucosal glands and intermittent expression on specific cells of mucosal epithelia but not on the ciliary process. * Expression more towards the apical side of the villi, # expression mainly on the epithelial brush border.

## Data Availability

Not applicable.
